# High frequency and molecular epidemiology of metallo-β-lactamase-producing gram-negative bacilli in a tertiary care hospital in Lahore, Pakistan

**DOI:** 10.1186/s13756-018-0417-y

**Published:** 2018-11-03

**Authors:** Noor Ul Ain, Anam Iftikhar, Syeda Sadia Bukhari, Samyyia Abrar, Shahida Hussain, Muhammad Hayat Haider, Farhan Rasheed, Saba Riaz

**Affiliations:** 1 Department of Microbiology and Molecular Genetics, University of the Punjab, Lahore, 5400 Pakistan; 2 Department of Pathology, Allama Iqbal Medical College, Lahore, Pakistan; 3 Citilab and Research Center, Lahore, Pakistan

## Abstract

**Background:**

Metallo-β-lactamase (MBL)-producing isolates have a strong impact on diagnostic and therapeutic decisions. A high frequency of MBL-producing gram-negative bacilli has been reported worldwide. The current study was based on determining the incidence of MBL-producing imipenem-resistant clinical isolates and investigating the β-lactamase gene variants in strains conferring resistance to a carbapenem drug (imipenem).

**Methods:**

A total of 924 gram negative isolates were recovered from a tertiary care hospital in Lahore, Pakistan, during a two-year period (July 2015 to February 2017). The initial selection of bacterial isolates was based on antibiotic susceptibility testing. Strains resistant to imipenem were processed for the molecular screening of β-lactamase genes. Statistical analysis for risk factor determination was based on age, gender, clinical specimen and type of infection.

**Results:**

The rate of imipenem resistance was calculated to be 56.51%. Among the 142 strains processed, the phenotypic tests revealed that the incidence of MBLs was 63.38% and 86.61% based on the combination disc test and the modified Hodge test, respectively. The frequencies of *bla*_TEM_, *bla*_SHV,_ *bla*_OXA,_ *bla*_IMP-1_, and *bla*_VIM_ genes were calculated to be 46%, 34%, 24%, 12.5% and 7%, respectively. The co-expression of *bla*_MBL_ (*bla*_IMP_ and *bla*_VIM_) and *bla*_ESBL_ (*bla*_TEM_, *bla*_SHV,_ *bla*_OXA_) was also detected through multiplex and singleplex PCR. *bla*_OXA_, *bla*_TEM_ and *bla*_SHV_ coexisted in 82% of the isolates. Co-expression of ESBL and MBL genes was found in 7% of the isolates.

**Conclusion:**

To our knowledge, this is the first report from Pakistan presenting the concomitant expression of *bla*_OXA_, *bla*_TEM_ and *bla*_SHV_ with *bla*_IMP-1_ and *bla*_VIM_ in MBL-producing gram-negative bacilli.

## Background

Dissemination of life-threatening infections caused by β-lactamase-producing pathogens is a major setback to antimicrobial therapy. The widespread use of carbapenems has resulted in the emergence of carbapenemases, conferring resistance against carbapenem drugs [1–3]. Resistance to carbapenems is worrisome because of the very limited therapeutic options available to treat resistant infections [4, 5]. The diverse mechanisms of resistance to imipenem include AmpC enzymes accompanied by membrane porin alterations and upregulation of efflux pumps [5, 6]. The second phenomenon is carbapenem hydrolysis by carbapenemases [7–9]. The epicentre for the emergence of carbapenemases and that of extended spectrum beta lactamases (ESBLs) were different, but the association of their genes is apparent through some studies, where ESBL genes are found to exist in MBL-producing isolates [10].

Among carbapenemases, metallo-β-lactamases (MBLs) are of prime importance for the region under study because of the emergence of new variants of MBL, such as New Delhi metallo-β-lactamase (NDM) [11] and various IMP variants from the subcontinent. MBLs belong to class B carbapenemases according to the Ambler classification system [12]. *bla*_IMP_, *bla*_NDM_ and *bla*_VIM_ are important MBL gene clusters that are carried by mobile plasmids compatible with a vast array of clinically important pathogens [13, 14]. In addition, oxacillinases belonging to class D include serine β-lactamases and are known to be associated primarily with *Enterobacteriaceae* carbapenem-resistant epidemics.

With more than 37 types of IMP carbapenemases known [15], IMP-1 was the first to be reported in Japan in 1991[16]. IMP-4-type enzymes, first discovered in Hong Kong during the 2000s [17], were later found to be responsible for an outbreak in 2005 in Melbourne, Australia [18]. The dissemination of resistance genes from *Serratia* spp. and *Pseudomonas aeruginosa* to other members of *Enterobacteriaceae* caused these genes to become endemic in Australia. Approximately 20 different subtypes of IMP enzymes have been described to be associated with *Pseudomonas* spp., *Acinetobacter* spp. and *Enterobacteriaceae* infections throughout the globe [19].

The first case of VIM-1 enzyme-conferred resistance was reported in 1999 in Verona, Italy [20]. With reports of VIM-2 being highly prevalent in Europe, Asia, America and Africa [21], a recent global surveillance study reported four new variants of VIM [22]. After their discovery in New Delhi in 2009 [23], NDM-1-producing *Klebsiella pneumoniae* and *E. coli* have been widely reported throughout the globe, including various European countries as well as China, Kenya, Japan, Algeria and Syria [24–27]. A research study has indicated dissemination of the NDM-1 carbapenemase gene through horizontal gene transfer in Pakistan, India and the UK [28].

The rise in carbapenem resistance in Asian countries has been evident, as reported imipenem resistance rose from 20% in the Philippines [29] to 40% in Vietnam [30]. Recently, > 50% carbapenem-resistant *Klebsiella pneumoniae* isolates have been recorded in India [31]. With the first report of emergence among *Acinetobacter baumannii* clinical isolates in Scotland in 1985 [32], plasmid-borne OXA-48 carbapenemases were reported to be highly disseminated in isolates from 18 countries in Europe and Africa [33].

The resistance pattern of metallo-β-lactamases in Pakistan has not been widely studied. In Pakistan, imipenem was only rarely available prior to the year 2000 [34]. Later, carbapenem resistance was reported mainly in *P. aeruginosa* in Lahore and Karachi [35, 36]. A report from Rawalpindi confirmed that 78% of isolates were MBL producers, with major incidence of MBL production in *Acinetobacter baumannii* and *Pseudomonas aeruginosa* [37].

A very limited number of reports have been published from Pakistan that are based on molecular analysis of genes acquired by carbapenem-resistant isolates. This study aimed to determine the incidence of MBLs through phenotypic and genotypic analyses. Moreover, this study is based on the detection of the gene variants responsible for resistance to carbapenem drugs. To the best of our knowledge, this is the first study from Lahore on the molecular epidemiology and coexistence of *bla*_ESBLs_ and *bla*_MBLs_.

## Methods

### Study design

Microbiological testing was conducted at the pathology laboratory of Allama Iqbal Medical College Lahore from July 2015 to July 2017. The study was approved by the Ethical Committee of Citilab and Research Centre Pakistan (CitiLab and Research Centre Ref # 30th − 15 CLRC/ 30th). The segment of the work based on molecular analysis was carried out at the Department of Microbiology and Molecular Genetics, University of the Punjab, Lahore.

### Bacterial isolates

A total of 3000 samples were obtained from clinical samples. All clinical specimens were subjected to isolation and identification of significant pathogens according to CLSI procedures (CLSI 2015, 2016). Among 2000 cultures with positive growth, 924 gram negative isolates bacilli were further screened for acquisition of imipenem resistance. On the basis of antibiotic susceptibility patterns, 142 isolates resistant to imipenem were further analysed by molecular tools. Identification of bacterial isolates was performed on the basis of culture characteristics, gram staining and conventional biochemical tests. Confirmation of gram-negative isolates was performed by API 20 NE identification strips (bioMerieux, France). The identified strains were stored in 30% glycerol broth at − 70 °C. Isolates were obtained from wound infections (*n* = 487), urine samples (*n* = 187), sputum samples (*n* = 90), tips and catheters (*n* = 47), fluids and effusions (*n* = 44) and others, including tissue samples, bone samples, and vaginal swabs (*n* = 57) (Table 3).

### Antimicrobial susceptibility testing

Antimicrobial susceptibility testing (AST) for all isolates was carried out by the Kirby-Bauer method [38] on Mueller-Hinton agar plates (Oxoid) as per Clinical and Laboratory Standard Institute (CLSI, 2016) recommendations. The antibiotic panel used in screening of the cultures was specific for gram-negative bacteria: penicillins (amoxicillin 30 μg, amoxicillin-clavulanic acid 40 μg, and piperacillin-tazobactam 30 μg), monobactam (aztreonam 30 μg), extended-spectrum cephalosporins (ceftriaxone 30 μg, cefepime 30 μg, cefotaxime 30 μg, cefoxitin 30 μg, and ceftazidime 30 μg), carbapenems (imipenem 30 μg), aminoglycosides (amikacin 30 μg and gentamicin 30 μg), quinolone (ciprofloxacin 30 μg) and trimethoprim-sulfamethoxazole (40 μg). The results of the susceptibility testing were used to calculate the multiple antibiotic resistance (MAR) index for the clinical isolates in order to estimate drug resistance trends and the emergence of new resistant isolates.

### Phenotypic detection of MBLs

Phenotypic detection of MBLs was based on three tests according to the CLSI guidelines (2015–2016). The combination disc test, using a disc of imipenem and imipenem with incorporated EDTA, was performed as per the method used by *Wadekar, Anuradha* [39]. A modified Hodge test (MHT) was performed according to the method used by Kumar et al. [40]. The results for each type of isolate were interpreted according to the criteria defined in CLSI 2016. All antibiotics were obtained from Oxoid, Inc. (Canada). E-strips with IMI and IMP/EDTA were used for epsilometer confirmation of MBLs according to the manufacturer’s instructions (Liofilchem®).

### Molecular characterization of MBLs

#### DNA template preparation for PCR

The template DNA was extracted from isolates using previously described methods [41]. Briefly, a single colony of bacterial isolate was immersed in low TE, and the suspension was boiled for 10 min. The bacterial cell emulsion was centrifuged, and DNA in the supernatant was directly used as a template for PCR amplification.

#### Detection of ESBL and MBL genes

All of the positive MBL isolates based on phenotypic detection (*n* = 123) were further confirmed by singleplex and multiplex colony PCR. Multiplex PCR for *bla*_TEM_, *bla*_OXA_ and *bla*_SHV_ detection was devised_._ The primer sequences used for the detection of *bla*_TEM_, *bla*_OXA_ and *bla*_SHV_ genes have been previously reported [42]. Screening for isolates having the *bla*_IMP-1_ gene and *bla*_VIM_ gene was performed by singleplex PCR using previously reported primers [43, 44]. The PCR reaction was set up with a 25 μl mixture containing 10X PCR buffer, 2.5 mM mixture of dNTPs, 20 pmol each primer and 2.5 U of Taq polymerase. The amplification conditions were set with an initial denaturation at 95 °C proceeded by 35 cycles of 1 min denaturation at 95 °C, 1.5 min annealing (temperatures mentioned in Table 1), extension for 1 min and final extension for 10 min at 72 °C. The Mg concentration was maintained between 1 and 1.5 mM.

**Table 1:** List of the Primers used for the detection of ESBL-Type variant (*bla*_OXA,_ *bla*_TEM_ and *bla*_SHV)_ and MBL-type variants (*bla*_IMP-1_ and *bla*_VIM_)

Primers	Sequences	Annealing temperature (Tm ^o^C)	References	Expected PCR product
*bla*_*IMP-1*_	AGCGCAGCATATTGATTGC ACAACCAGATGCTGCCTTACC	53.6	[43]	587
*Bla*_VIM_	ATGGTCGTTATGGCATATC TGGGCCGTGTCAGCCAGAT	57	[44]	510
*Bla*_TEM_	CCCCGAAGAAGTCCTTTC ATCAGCAATAGTCCCAGC	55	[42]	500
*Bla*_SHV_	AGGGCTTGACTGCCATTTTG ATTTGCGTGATTTCATTT	55	[42]	400
*Bla*_OXA_	ATATCTCGCTTGTTGCATCTCC AAACCCTTCAGCTCATCC	55	[42]	600

#### Statistical analysis

The collected demographic data were statistically analysed using the Statistical Package for Social Sciences (SPSS version 23). The proportions of *Acinetobacter* spp*., Pseudomonas* spp. and members of *Enterobacteriaceae* were calculated using the chi-square test and odds ratios (ORs). A *p* value of < 0.05 was considered statistically significant. The associations among the type of infection, age, gender and type of isolate were calculated.

## Results

### Distribution of clinical isolates

In this study, the resistance pattern of imipenem-resistant clinical isolates was assessed, and the incidence of MBL production among these isolates was determined. Moreover, the significant gene variants associated with the MBL phenotype were analysed. Demographic factors and sites of infection were major highlights of the statistical data analysis. Out of a total of 942 isolates, the total frequency of imipenem resistance was calculated to be 56.512% (*n* = 512). The highest resistance to imipenem was observed for *Acinetobacter* spp., at 61.89%. *Pseudomonas* spp. ranked second in terms of the acquisition of imipenem resistance, with a frequency of 61.89%, followed by *Klebsiella* spp. (50.26%) and *Escherichia coli* (37.97%). Males were found to be more prone to the acquisition of imipenem-resistant infections (60.69%) compared to females (39.31%). The infectivity rates varied between different age groups, with the maximum mean observed among individuals of the age group 20–40 years. The mean age of individuals acquiring MBL infection was 30 years. Wound infections were found to be the most dominant type of infection (51.70%), followed by urinary tract infections (19.86%), respiratory tract infections (9.6%), and infections associated with indwelling catheters (5.2%).

### Antibiotic susceptibility testing of MBL isolates

The panel of antibiotics recommended according to CLSI 2016 guidelines was applied for all isolates belonging to *Enterobacteriaceae, Pseudomonas* spp. and *Acinetobacter* spp. *Escherichia coli* and *Klebsiella* spp. exhibited susceptibility to gentamycin and piperacillin-tazobactam (23%), amikacin (16%) and sulfamethoxazole/trimethoprim (15%). All the generations of cephalosporins, carbapenem and monobactams showed a complete resistance pattern. *Pseudomonas* spp. and *Acinetobacter* spp. presented susceptibility to amikacin and aztreonam (12%), piperacillin-tazobactam (8%), sulfamethoxazole/trimethoprim and gentamycin (7%) and ciprofloxacin (3%). Multiple antibiotic resistance (MAR) index values for > 50% of the isolates fell in the range of 0.81–1.00, and 86% of the *Pseudomonas* spp. isolates fell in the range of 0.91–1.0. *E. coli* predominantly had a MAR index value of 0.91–1. A total of 75% of the *Klebsiella* spp. isolates had a MAR index value ranging between 0.81 and 1.

### Phenotypic detection of MBLs

Out of the 906 isolates analysed, 142 randomly selected isolates were suspected to produce metallo-β-lactamases. Among these isolates, 63.38% (*n* = 90) revealed a positive combination disc test, whereas 36.61% (*n* = 52) remained non-determinable by CDST. A total of 86.61% (*n* = 123) of the isolates were confirmed to be MBL producers through the modified Hodge test with meropenem. However, the modified Hodge test with imipenem detected 78.17% (*n* = 111) of the isolates as positive for MBL production and 22.53% (*n* = 32) as negative for MBL production. A total of 68% (*n* = 96%) of the strains were confirmed to exhibit the MBL phenotype through the epsilometer test (E-test).

### Multiplex PCR for *bla*_OXA_, *bla*_TEM_, *bla*_SHV,_*bla*_IMP_ and *bla*_VIM_

**Fig. 1:**
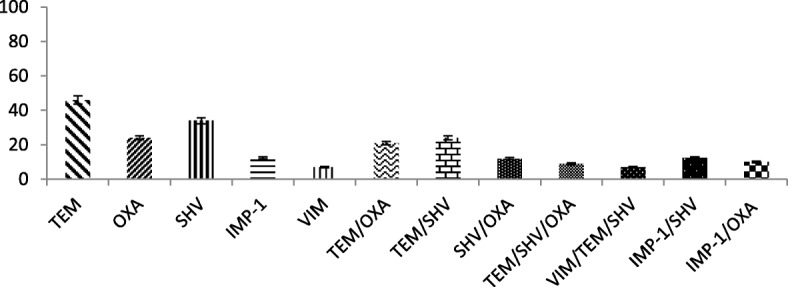
Frequencies of different gene variants (*TEM, SHV, OXA, VIM, IMP*) in MBL producing clinical isolates. The presence of genes *bla*_OXA_, *bla*_TEM_, *bla*_SHV_ *bla*_IMP-1_and bla_*VIM*_was detected in MBL strains through PCR

The presence of *bla*_OXA_, *bla*_TEM_ and *bla*_SHV_ genes was confirmed in 57.74% (*n* = 82%) of MBL-producing strains by multiplex PCR. The existence of the *bla*_TEM_ gene in MBL-producing isolates was found to be the most prevalent, at 46%, followed by the *bla*_SHV_ gene (34%) and *bla*_*OXA*_ gene (24%). *bla*_IMP-1_ and bla_*VIM*_ genes were detected in 12.5% (*n* = 18) and 7% (*n* = 10) of strains, respectively. The coexistence of all these genes was determined by multiplex PCR. In total, 60% (49/82) of the MBL-positive strains were found to have the *bla*_OXA_, *bla*_TEM_ and *bla*_SHV_ genes in coexistence with each other. The *bla*_TEM_ gene was found to coexist with *bla*_*OXA*_*-type* variants in 21% (*n* = 30) of the MBL producers. The combination of *bla*_TEM_ and *bla*_SHV_ was detected to be the most common, as exhibited by 24% of the strains. The coexistence of *bla*_OXA_ and *bla*_SHV_ genes was observed in 12% of the total isolates. The three genes *bla*_TEM,_ *bla*_OXA_ and *bla*_SHV_ were found to coexist in 9% of the strains (Fig. 1).

#### Statistical analysis

Statistical analysis was performed with SPSS version 23.0 individually for all groups of isolates, including *Escherichia coli*, *Klebsiella* spp., *Acinetobacter* spp. and *Pseudomonas* spp. (Table 2). The associations among demographic variables, including age, gender and type of infections, were determined by calculating odd ratios and performing the chi-square test. A *p*-value of < 0.05 was considered statistically significant (Tables 2, 3, 4).

**Table 2:** Comparison of Carbapenem resistant isolates between different age groups

Age (years)	*No.* of Isolates	Imipenem		Chi value	OR (95% CI)	*p*-value
		R	S			
*Escherchia coli*						
1–9	5	4 (80%)	1 (20%)	–	2.28 [0.23–22.87]	0.643
10–19	7	4 (57.14%)	3 (42.85%)		0.758 [0.157–3.66]	0.704
20–29	38	15 (39.47%)	23 (60.52%)	10.10	0.317 [0.153–0.657]	0.001
30–39	29	9 (31.03%)	20 (68.96%)	7.85	0.308 [0.132–0.723]	0.005
40–49	34	13 (38.23%)	21 (61.76%)	2.45	0.537 [0.245–1.176]	0.117
50–59	32	11 (34.37%)	21 (65.62%)	4.58	0.414 [0.182–0.940]	0.032
60–69	26	8 (30.76%)	18 (69.23%)	5.69	0.316 [0.120–0.831]	0.017
70–79	9	5 (55.55%)	4 (44.44%)	–	0.809 [0.177–3.69]	1.000
> 79	7	2 (28.57%)	5 (71.42%)	–	0.150 [0.018–1.237]	0.145
*Acinetobacter* spp.						
1–9	11	8 (66.66%)	4 (33.33%)	1.252	1.8 [0.638–5.072]	0.999
10–19	25	18 (72%)	7 (28%)	3.14	2.015 [0.92–4.41]	0.264
20–29	39	29 (74.35%)	10 (25.64%)	8.05	2.94 [1.37–6.33]	0.076
30–39	42	31 (73.80%)	11 (26.19%)	0.56	1.33 [0.633–2.79]	0.004
40–49	38	21 (55.26%)	17 (44.74%)	6.18	2.94 [1.28–6.73]	0.451
50–59	34	24 (70.58%)	10 (29.41%)	–	3.87 [1.26–11.81]	0.009
60–69	20	15 (75%)	5 (25%)	–	1.031 [0.23–4.53]	0.026
70–79	10	6 (60%)	4 (40%)	–		0.999
> 79	4	4 (100%)	0 (0%)			
*Klebsiella* spp.						
1–9	11	6 (54.54%)	5 (45.45)	0.870	0.505 [0.119–2.145]	0.351
10–19	10	6 (60%)	4 (40%)	–	0.857 [0.220–3.343]	1.000
20–29	40	22 (55%)	18 (45%)	1.040	0.695 [0.345–1.401]	0.824
30–39	41	19 (46.34%)	22 (53.65%)	1.52	0.645 [0.320–1.299]	0.218
40–49	26	15 (57.69%)	11 (42.30%)	0.749	1.458 [0.619–3.43]	0.387
50–59	26	8 (30.76%)	18 (69.23%)	5.230	0.356 [0.143–0.883]	0.022
60–69	19	9 (47.36%)	10 (52.63)	0.097	0.851 [0.309–2.343]	0.755
70–79	11	8 (72.72%)	3 (27.27%)	–	2.286 [0.493–10.605]	0.466
> 79	5	2 (40%)	3 (60%)	–	0.417 [0.051–3.435]	0.608
*Pseudomonas* spp.						
1–9	9	6 (66.67%)	3 (33.34%)	–	1.05 [0.22–5.13]	1.000
10–19	34	20 (58.83%)	14 (41.17%)	0.497	0.71 [0.28–1.82]	0.481
20–29	75	54 (72%)	21 (28%)	5.093	2.02 [1.09–3.74]	0.024
30–39	61	38 (62.29%)	23 (37.70%)	2.127	1.59 [0.85–3.00]	0.145
40–49	44	23 (52.27%)	21 (47.72)	0.130	1.14 [0.56–2.31]	0.718
50–59	51	30 (58.83%)	21 (41.17%)	1.917	1.63 [0.81–3.25]	0.166
60–69	25	14 (56%)	11 (44%)	0.410	1.35 [0.54–3.41]	0.522
70–79	6	3 (50%)	3 (50%)	–	0.63 [0.11–3.66]	0.670
> 79	2	2 (100%)	0	–	–	0.477

*OR Odd ratio&** *p* value < 0.05 is considered as statistically significant

**Table 3:** Association of imipenem resistant isolates with type of clinical specimens

Isolate	Sample	(*N*)	Imipenem		Chi value	OR	*p*-value
			R	S			
*Escherichia coli*	Wound	65	22	43	23.013	0.276 [0.159–0.478]	0.000
	Fluids & Effusion	13	3	10	–	0.123 [0.027–0.553]	0.007
	Tips & Catheters	6	5	1	–	1.833 [0.192–17.48]	1.00
	Urine	79	34	45	0.894	0.756 [0.422–1.351]	0.344
	Sputum	12	5	7	0.096	0.824 [0.242–2.806]	0.757
	Others	14	4	10	–	0.403 [0.290–0.559]	0.070
*Acinetobacter* spp.	Wound	130	92	38	7.16	1.79 [1.16–2.75]	0.006
	Fluids & Effusion	13	11	2	–	6.67 [1.26–35.28]	0.021
	Tips & Catheters	22	17	5	0.171	1.32 [0.35–4.97]	0.679
	Urine	21	11	10	0.265	1.27 [0.51–3.15]	0.606
	Sputum	25	15	10	2.73	2.17 [0.85–5.50]	0.098
	Others	13	10	3	–	4.38 [1.05–18.17]	0.056
*Klebsiella* spp.	Wound	71	43	28	0.004	0.983 [0.59–1.64]	0.947
	Fluids & Effusion	11	6	5	0.031	0.88 [0.22–3.49]	0.861
	Tips & Catheters	10	7	3	–	0.75 [0.16–3.52]	0.700
	Urine	58	25	33	0.534	0.79 [0.43–1.48]	0.465
	Sputum	30	13	17	0.110	0.86 [0.36–2.06]	0.740
	Others	10	4	6	–	0.59 [0.15–2.35]	0.504
*Pseudomonas* spp.	Wound	221	142	79	1.84	1.29 [0.89–1.85]	0.174
	Fluids & Effusion	7	5	2	–	2.12 [0.36–12.38]	0.680
	Tips & Catheters	9	6	3	–	0.62 [0.13–2.99]	0.674
	Urine	29	19	10	4.669	2.44 [1.06–5.58]	0.031
	Sputum	23	9	14	0.547	0.69 [0.27–1.81]	0.459
	Others	20	11	9	0.210	1.29 [0.43–3.84]	0.647

*OR Odd ratio&** *p* value < 0.05 is considered as statistically significant

**Table 4:** Comparison of infection rate between male and female

Isolates	Gender	(*N*)	Imipenem		Chi value	OR*	*p*-value**
			R	S			
*Escherichia coli*	Male	106	41	65	0.0067	1.025 [0.57–1.85]	0.934
	Female	84	32	52			
*Acinetobacter* spp.	Male	136	94	42	41.05	4.51 [2.81–7.24]	0.0001
	Female	90	63	127			
*Klebsiella* spp.	Male	97	57	40	4.67	1.87 [1.058–3.328]	0.03
	Female	95	41	54			
*Pseudomonas* spp.	Male	203	125	78	0.0017	0.98 [0.61–1.59]	0.483
	Female	110	68	42			

*OR Odd ratio&** *p* value < 0.05 is considered as statistically significant

## Discussion

Pakistan is a country where empirical drug therapy and misuse of antibiotics are common practice. Poor sanitation, filthy practices in clinical settings, and ill-informed health care workers are factors in the dissemination of nosocomial pathogensto the community. Routinely used second-generation drugs are quickly being replaced by drugs of last resort, and this situation is ultimately an enduring threat to mankind. According to one estimate, first-line antibiotic-resistant pathogens account for 25,692 neonatal deaths annually in Pakistan [45]. Resistance to carbapenems has been significantly observed in Asian countries, including Pakistan [30, 46, 47]. At present, numerous reports of MBL producers and ESBL producers from Pakistan present clinical catastrophes and alarming health issues [41, 48, 49]. The present study demonstrates the frequency of imipenem resistance among clinical isolates and the incidence of MBLS producers associated with imipenem resistance in connection with various demographic factors and types of infection. The frequency of imipenem resistance in our study was 56%, which is significantly higher than the data reported from Asian countries in the last decade (2002–2012), with one epidemiological study reporting 1.9% resistance to imipenem and 2.4% resistance to meropenem [4].

Comparison of the antibiotic resistance profiles of all the pathogens with those reported in other recent studies has revealed relatively similar patterns. The resistance pattern of *Acinetobacter* spp. to imipenem in our study (61.89%) is in conformity with reports by Anwar et al., but a lower rate (77.5%) of imipenem resistance in this region was reported by Shamim et al. [50]. *Acinetobacter* spp., the pathogen renowned for hospital-acquired infections, was predominantly found to be associated with wound infections and was the causative agent of 92% infections associated with imipenem resistance. *Pseudomonas* spp. was the second most prominent pathogen associated with the acquisition of imipenem resistance, with a frequency of 61.89%, and has been noted in other reports from Pakistan, presenting imipenem resistance rates of 13.42% in 2011 and of 28% and 49.5% in 2015, thus demonstrating a sharp rise in the frequency of imipenem resistance [49, 51, 52]. The victims of *Pseudomonas* spp. infections were found predominantly in the group of patients with post-burn infections. This finding is in conformity with studies on invasive burn wound infections that document *P. aeruginosa* as a leading pathogen among gram-negative organisms [53, 54]. Wound infections were particularly found to be associated with *Acinetobacter* spp. (OR = 1.79 [1.16–2.75]) and *Pseudomonas* spp. *OR = 1.29 [0.89–1.85]).

The incidence of MBL among imipenem-resistant *Acinetobacter* spp. was calculated to be 89%, which is comparable to that reported in a study by Anwar et al., presenting a frequency of 83.3% MBLs among carbapenem-resistant isolates [55]. A total of 78% of the imipenem-resistant *Pseudomonas* spp. isolates were detected to be MBL producers by the MHT test, representing a lower incidence compared to the study by Shan et al. that stated the incidence rate to be 87.5% [49].

Our study demonstrates the OXA-type variants to be predominantly associated with resistance to imipenem. All of the isolates harbouring *bla*_IMP_ were also found to harbour *bla*_TEM_. Despite the uncommon origin of two major groups of β-lactamases, *bla*_ESBL_ and *bla*_MBL,_ their association is imminent according to recent research [10]. We analysed the isolates for the coexistence of MBL-type and ESBL-type variants. The *bla*_VIM_ gene variant for MBL production was also found to be in coexistence with *bla*_TEM_ and *bla*_SHV_ in *Providencia stuartii* and *Enterobacter* spp. A total of 12.5% isolates were found to coexhibit the *bla*_IMP-1_ and *bla*_SHV_ genes, whereas 10% were positive for the *bla*_*OXA*_ gene along with the *bla*_IMP-1_ gene.

The abovementioned mechanism of coexistence of genes has been reported in *Klebsiella pneumoniae, Escherichia coli, Salmonella* spp*.* and *Enterobacter* spp. [56]*.* Plasmids containing the *bla*_NDM-1_ gene have been observed to coexhibit genes for CTX-M, TEM-1 and OXA-1 enzymes. Major ESBL and MBL genes, including *bla*_CTX-M_, *bla*_SHV_, *bla*_TEM_, and *bla*_OXA-51_, and genes for the VIM-family and IMP-family, have been reported to coexist in clinically resistant *Acinetobacter baumannii* in Iran [57]. However, one study concluded that there was no significant relationship between ESBL and MBL production genes [57]. Ertapenem-resistant, ESBL-producing *Klebsiella pneumoniae* isolates have been reported in Italy and shown to carry novel porin variants that contributed to the reduced susceptibility of isolates to meropenem and imipenem [58].

The coexistence of the genes for MBL and ESBL variants in our isolates indicates the simultaneity of the emergence of different variants of β-lactamases among pathogens in our clinical settings. This finding also suggests that the resistance against imipenem in our isolates is mediated by MBL-type enzymes along with the overproduction of ESBL-type enzymes, as suggested by other studies [5, 7]. To the best of our knowledge, this is the first study from Pakistan reporting the coexistence of *bla*_IMP_ with *bla*_TEM_-type and *SHV*-type variants. None of the isolates was found to coexhibit all the tested genes (*bla*_OXA_, *bla*_TEM_, *bla*_SHV_, *bla*_IMP-1_ and *bla*_VIM_).

## Conclusion

In conclusion, ESBL- and MBL-producing bacterial isolates are emerging very rapidly in the region. A great number of carbapenem-resistant clinical bacterial species are resistant to most of the commonly used antibiotics, demonstrating the rise of super-bacteria and their pan-resistance to antimicrobial therapy. Determining the resistance mechanisms and the root cause for their elimination are of great importance. It is also important to implement the routine screening of ESBLs and MBLs in laboratory procedures before antibiotic therapy begins. Further studies are required to specify other types of gene variants prevalent among clinical isolates in our region for the implication of medication in clinical settings.

## Abbreviations

CDST: Combination disc test
DDST: Double disc synergy test
E test: Epsilometer test
ESBLs: Extended-spectrum β-lactamase-producing strains
ESBLs: Extended-spectrum β-lactamases
MBLs: Metallo-β-lactamases
MDR: Multidrug-resistant
MIC: Minimum inhibitory concentration
